# Expected Duration of Adverse Pregnancy Outcomes after Zika Epidemic

**DOI:** 10.3201/eid2401.170482

**Published:** 2018-01

**Authors:** Rosalind M. Eggo, Adam J. Kucharski

**Affiliations:** London School of Hygiene & Tropical Medicine, London, UK

**Keywords:** Zika virus, viruses, vector-borne infections, fetal development, surveillance, monitoring, epidemiology, Brazil, pregnancy, miscarriage, microcephaly

## Abstract

Evidence is increasing that Zika virus–related adverse outcomes can occur throughout pregnancy. Mathematical modeling analysis using reported outcome data suggests that surveillance for these outcomes should begin as soon as an outbreak is detected and should continue for 40 weeks after the outbreak ends.

Quantifying the risk for adverse pregnancy outcomes (APOs) after Zika virus infection is of critical public health importance. Recent studies have suggested that risk for microcephaly is concentrated in pregnancies in which infection occurs during the first trimester (*1*,*2*). However, microcephaly is at the severe end of the APO spectrum and might have a different risk profile from other outcomes: brain abnormality and malformation, eye anomalies, neural tube defects, arthrogryposis, congenital deafness, and others (*3*). In particular, estimates of APOs after symptomatic confirmed Zika virus infection suggest risk for fetal injury throughout pregnancy (*3*,*4*). Thus, a better understanding of the likely duration and risk for APOs after Zika virus outbreaks is urgently needed (*2*). We used surveillance and clinical data to estimate the timing and number of expected APO events after observed Zika outbreaks in 9 regions of Brazil during April 2015–July 2017.

## The Study

To quantify APO risk, we used data from a study that recruited 345 pregnant women with rash in the previous 5 days, of whom 134 tested positive for Zika virus infection (*4*). Excluding 9 losses to follow-up, we followed a cohort of 125 women during pregnancy; surviving infants were examined for APOs. A total of 58 APOs occurred in this group; microcephaly was infrequent (4 [6.9%] of 58) but severe (*4*).

**Figure 1:**
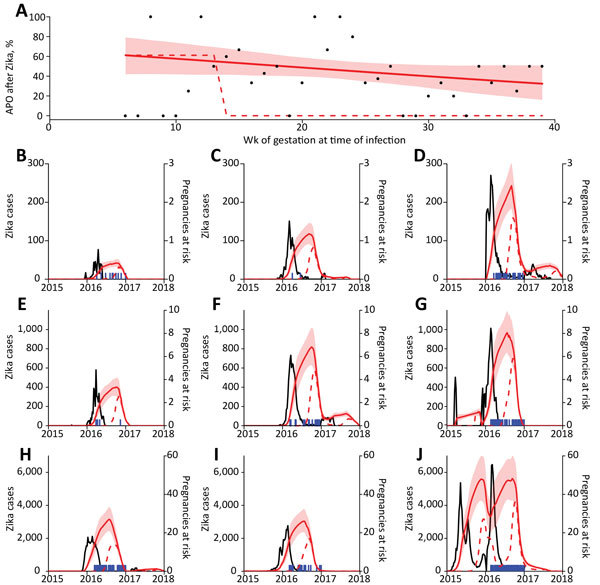
Relationship between Zika virus infection and expected related APOs per 1,000 pregnancies in Brazil during April 2015–July 2017. A) Percentage of APOs (fetal loss at any gestational age, stillbirth, neonatal abnormality) given symptomatic PCR-confirmed Zika virus infection. Points show weekly proportion with APO (*4*); red line indicates fit to data with a generalized linear model, and shading indicates 95% CIs; dashed line indicates fixed risk in first trimester only (*5*). B–J) Blue lines indicate suspected Zika cases in different regions; red lines indicate expected number of births with Zika-associated APO in subsequent weeks based on the 2 risk distributions in panel A. Shaded regions indicate 95% CIs. Model assumes 17% of Zika virus infections are reported (*5*,*6*). APO, adverse pregnancy outcome.

We fitted a logistic model to individual-level data for the 125 followed-up women to estimate the proportion of APOs after symptomatic Zika virus infection at each week of gestation (Figure 1, panel A). Although the fitted linear model suggested a decline in risk over time, the model did not perform significantly better than a model with constant risk for APO at any gestational age (Technical Appendix). For comparison, we also considered a theoretical risk profile in which APO risk occurs only during the first trimester (Figure 1, panel A).

We used these risk profiles to estimate the period through which an elevated rate of APOs would be expected after the 2015–2016 Zika epidemic in 9 regions of Brazil (Figure 1, panels B–J). We superimposed the timing of confirmed microcephaly cases in each region to assess the relationship between observed microcephaly and expected duration of elevated APO risk but did not fit explicitly to microcephaly incidence data. If risk were assumed to occur only during the first trimester, the period of APOs would be shorter than the duration of observed microcephaly events. In contrast, the predicted durations of APOs based on risk throughout pregnancy were more consistent with the observed distribution of microcephaly in these regions.

**Figure 2:**
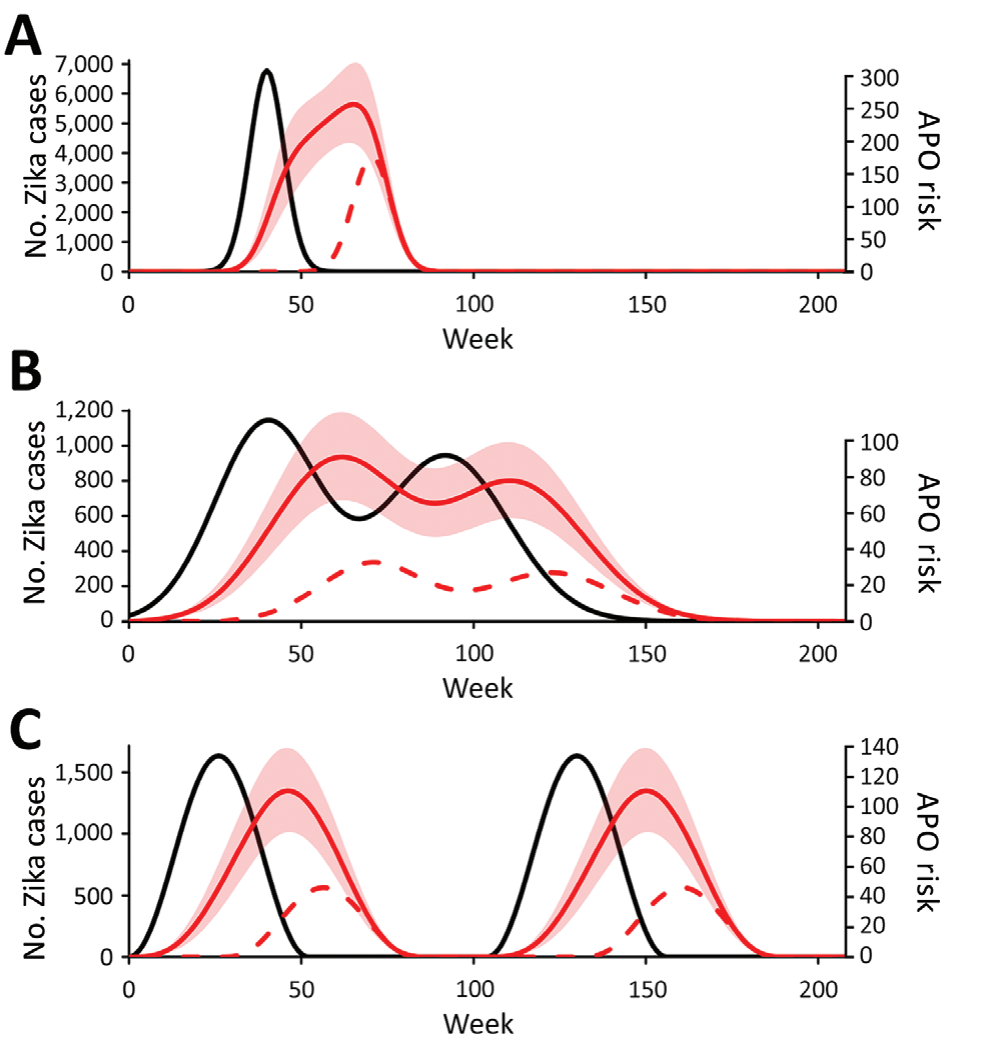
Expected temporal distribution of Zika virus–related adverse pregnancy outcomes under different hypothetical outbreak scenarios, Brazil, April 2015–July 2017. Black lines indicate Zika cases; red lines indicate risk (APOs/1,000 births) for Zika-associated APO in subsequent weeks based on the 2 risk distributions in panel A. Dashed lines indicate timing of outbreaks: A) short, single-peaked outbreak; B) double-peaked outbreak; C) biennial epidemics (i.e., a seasonal endemic state). A population size of 1 million, reporting of 17% of Zika infections, and a 50% attack rate during a 4-year period were assumed. APO, adverse pregnancy outcome.

To examine the potential duration and risk for Zika-associated APOs more generally, we also predicted the pattern of APOs under 3 hypothetical epidemic scenarios: single outbreak, multipeaked epidemic, and endemic transmission (Figure 2). For each epidemic scenario, the model suggested that the duration of elevated risk was much longer than the duration of cases if APOs could occur from infection in any gestational week. This observation means that in areas where seasonal outbreaks of Zika occur, the risk for APOs might not return to baseline levels between epidemics, and Zika-specific interventions based on timing of pregnancy might be less effective (*7*).

Our findings are subject to several limitations. First, we based the estimation of APO risk by gestation period on a cohort study of symptomatic infection with rash, which does not occur with all Zika virus infections (*8*). However, recent evidence suggests the risk for APOs is similar for symptomatic and asymptomatic infection (*9*). We included pregnancy loss during the first trimester (miscarriage) as an APO, but excluding these 5 cases did not alter the findings (online Technical Appendix, Sensitivity Analysis on Inclusion of Miscarriages section). Moreover, evidence suggests that APOs might not be detectable at birth but appear later, which would underestimate the frequency of APOs (*10*).

Second, the data were from patients recruited in Rio de Janeiro, whereas we considered potential risk across all regions of Brazil. Although the cohort was large and APO data detailed, numbers of exposed women in each gestational week were low, leading to large CIs on the risk profile (Figure 1, panel A). We therefore used a linear model to estimate the risk at each gestational week because data were insufficient to fit a more complex risk function. The range of data (6–39 weeks’ gestation) also constrained our estimates.

Third, publicly available epidemiologic reports from Brazil recorded microcephaly cases, rather than all forms of APO. We qualitatively compared these microcephaly reports with our estimates for the duration of risk for APOs, but the risk for microcephaly by gestational week might differ from the overall risk for APOs. Different regions are likely to have differing baseline levels of APOs in the absence of Zika virus infection; we therefore focused our analysis on the risk for APOs associated with Zika virus infection. Why some areas of Latin America have reported more cases of microcephaly than others remains unclear (*11*). There may be unmeasured cofactors that alter the risk for APO on Zika virus infection (*12*). Another factor could be differences in the proportion of Zika cases reported, which could lead to variation in incidence of APOs. We assumed 17% of Zika infections were reported (*6*,*8*); if the proportion reported was larger, it would mean fewer women were infected during the epidemic, and hence fewer would be expected APOs (Technical Appendix, Sensitivity Analysis on Fraction of Cases Reported section).

Finally, Brazil made Zika notifiable in November 2015, which might have increased reporting (*13*). In addition, the Zika incidence data varied markedly by region, which may be due to true differences in outbreak dynamics or to differences in reporting of cases (Figure 1). Although variability in weekly Zika incidence data would alter the precise relationship between Zika cases and population-level rate of APO, the general shape and duration of enhanced risk estimated in the model remains the same (online Technical Appendix, Sensitivity Analysis on Fraction of Cases Reported section).

## Conclusions

Our results suggest that if fetal injury from Zika virus infection can occur across a range of gestational ages, APOs after a Zika outbreak could occur for a long time after the outbreak subsided. This duration is longer than if the risk is assumed to be in the first trimester only (*2*,*14*). Combined with epidemiologic reports of APOs collected in Brazil, which show an increase in microcephaly rate at a time inconsistent with first trimester–only risk, evidence is mounting to recommend extended surveillance for APOs and to include a spectrum of outcomes, not only microcephaly (*10*,*15*).

Our results suggest that when Zika outbreaks are identified, surveillance and planning for infection-associated APOs might need to focus on a longer period than previously thought. In addition to the potential for APOs several months after an epidemic, the risk period may begin soon after the outbreak is detected. Further studies are crucial to refine the risk for APO during gestation and to ensure pregnant women can be correctly informed of their risk, so that population-level surveillance can be effectively implemented.

Technical Appendix Additional methods for the study on determining the expected duration of adverse pregnancy outcomes after Zika epidemic, Brazil, April 2015–July 2017.
