# Supplementary material for: Expected Duration of Adverse Pregnancy Outcomes after Zika Epidemic
Source: Emerg Infect Dis. 2018 Jan;24(1):127–30. doi: 10.3201/eid2401.170482 (PMC5749473; doi:10.3201/eid2401.170482)
Supplement: Technical Appendix — Additional methods for the study on determining the expected duration of adverse pregnancy outcomes after Zika epidemic, Brazil, April 2015–July 2017. [file 17-0482-Techapp-s1.pdf]

Article DOI: https://doi.org/10.3201/eid2401.170482

Expected Duration of Adverse Pregnancy Outcomes After Zika Epidemic

Technical Appendix

Data

Data were drawn from Brasil et al. (*1*) (Technical Appendix Table 1). We included cases of fetal loss in the first trimester (miscarriage) in the analysis but include sensitivity analysis on this in the Sensitivity Analysis on Inclusion of Miscarriages section.

Zika Incidence

Time series of the weekly number of Zika cases in each region were initially drawn from the supplement of Ferguson et al. (*2*) and then updated with more recent values by the same methods, i.e., transcribed from situation reports in each affected region. Data from Bahia are updated in line with surveillance reports. All values are provided in a public github repository (https://github.com/rozeggo/microcephaly_Brazil).

Analysis Code

All code for the analyses conducted here are provided in https://github.com/rozeggo/microcephaly_Brazil.

Comparison of Fitted Model and Constant Risk

We estimated the adverse pregnancy outcome (APO) risk function by fitting a linear model with binomial link function to the individual-level APO data using the R package "*mgcv*" and function "glm()." In the model, the intercept was estimated to be 0.6685 (SE 0.5163), and the estimated coefficient for risk for APO by week of gestation was –0.0360 (SE 0.0215). Although it has previously been suggested that there is a higher risk for Zika-associated adverse

outcomes earlier in pregnancy, the performance of the linear model was not substantially better than a simpler model that assumed a constant risk across the whole pregnancy period (model with linear term had Akaike Information Criterion, AIC = 173.76; model with constant risk had AIC = 174.64).

Using Case Data to Estimate Pregnancies with APO

We first calculated total number of reported cases, *C*, during the epidemic:

$$C = \sum_{t} c_t$$

where c_t is the number of cases reported in week *t*. We combined this value with the assumed proportion of cases that were reported, *r*, and the population size *N* to calculate the overall attack rate, *A*, for the population:

$$A = \frac{C}{rN}$$

In our main analysis, we assumed $r = 0.17$, based on the estimated number of case-women who attended health care facilities (C = 30,000), population size (N = 275,000) and postepidemic seroprevalence (A = 0.66) in the 2013–14 Zika outbreak in French Polynesia (*3*,*4*), but tested this assumption in a sensitivity analysis (see Sensitivity Analysis on Fraction of Cases Reported section.

To estimate weekly expected number of pregnancies with APO, we estimated the probability that someone would have been infected during their pregnancy. For a woman who was 6 weeks pregnant in week *t* of the outbreak (we did not consider the earlier period of pregnancy because no APO data were available for this (Figure 1, panel A), the risk for APO, a_t, at *t* weeks was:

$$a_t = \sum_{j=t}^{t+39-6} \frac{c_t}{C} f(j - t + 6)$$

where *f(x)* denotes the risk for APO given infection in gestation week *x*, and we assume a gestation period of 39 weeks. These women would therefore be expected to give birth in week *t*+39–6. Finally we scaled the risk by the number of pregnant women expected each week;

because the birth rate was 14 per 1,000 in 2015 (*5*), we would expect (14 × N)/(52 × 1,000) births per week.

The calculations in the simulation study were performed in the same way, except with simulated trajectories for the number of cases over time.

Sensitivity Analysis on Fraction of Cases Reported

To determine the effect on our estimates of assuming 17% of Zika cases are reported, we also examined the findings when we assumed 40% of Zika cases are reported (Technical Appendix Figure 1). The overall number of pregnancies at risk for APO is lower, the time period of elevated risk is the same, and therefore the overall public health message is unchanged by this assumption (Technical Appendix Figure 2).

Sensitivity Analysis on Inclusion of Miscarriages

To test the effect of first trimester fetal loss (miscarriage in Brasil et al. [*1*]) we refitted the model with those 5 events excluded, and show the same results as in the main paper (Technical Appendix Table 2, Technical Appendix Figure 3). There is little effect on estimates: intercept = 0.2427 (SE 0.5538), week coefficient = –0.0206 (SE 0.0226).

References

1. Brasil P, Pereira JP Jr, Moreira ME, Ribeiro Nogueira RM, Damasceno L, Wakimoto M, et al. Zika virus infection in pregnant women in Rio de Janeiro. N Engl J Med. 2016;375:2321–34. PubMed http://dx.doi.org/10.1056/NEJMoa1602412

2. Ferguson NM, Cucunuba ZM, Dorigatti I, Nedjati-Gilani GL, Donnelly CA, Basanez M-G, et al. Countering the Zika epidemic in Latin America. Science. 2016;353:353–4. http://dx.doi.org/10.1126/science.aag0219

3. Kucharski AJ, Funk S, Eggo RM, Mallet H-P, Edmunds WJ, Nilles EJ. Transmission dynamics of Zika virus in island populations: a modelling analysis of the 2013–14 French Polynesia outbreak. PLoS Negl Trop Dis. 2016;10:e0004726. PubMed http://dx.doi.org/10.1371/journal.pntd.0004726

4. Cauchemez S, Besnard M, Bompard P, Dub T, Guillemette-Artur P, Eyrolle-Guignot D, et al. Association between Zika virus and microcephaly in French Polynesia, 2013–15: a retrospective study. Lancet. 2016;387:2125–32. PubMed http://dx.doi.org/10.1016/S0140-6736(16)00651-6

5. World Bank. Birth rate, crude (per 1,000 people) [cited 2017 Mar 23]. https://data.worldbank.org/indicator/SP.DYN.CBRT.IN

Technical Appendix Table 1. Pregnancy and Zika virus APO data that include first-trimester fetal loss (miscarriage), Brazil, April 2015–July 2017

Week of gestation	APO negative	APO positive	Total pregnancies
6	1	2	3
7	1	0	1
8	0	2	2
9	1	1	2
10	2	1	3
11	3	2	5
12	0	2	2
13	1	1	2
14	2	3	5
15	1	2	3
16	2	1	3
17	4	3	7
18	3	3	6
19	4	0	4
20	2	1	3
21	0	4	4
22	2	4	6
23	0	4	4
24	1	4	5
25	4	2	6
26	5	3	8
27	3	3	6
28	2	0	2
29	2	0	2
30	4	1	5
31	2	1	3
32	4	1	5
33	1	0	1
34	2	2	4
35	2	1	3
36	1	1	2
37	3	1	4
38	1	1	2
39	1	1	2
Total	67	58	125

*Data are from Brasil et al. (*1*). APO, adverse pregnancy outcome.

Technical Appendix Table 2. Pregnancy and Zika virus APO data that exclude first-trimester fetal loss (miscarriage), Brazil, April 2015–July 2017

Week of gestation	APO negative	APO positive	Total pregnancies
6	1	0	1
7	1	0	1
8	0	2	2
9	1	0	1
10	2	0	2
11	3	1	4
12	0	2	2
13	1	1	2
14	2	3	5
15	1	2	3
16	2	1	3
17	4	3	7
18	3	3	6
19	4	0	4
20	2	1	3
21	0	4	4
22	2	4	6
23	0	4	4
24	1	4	5
25	4	2	6
26	5	3	8
27	3	3	6
28	2	0	2
29	2	0	2
30	4	1	5
31	2	1	3
32	4	1	5
33	1	0	1
34	2	2	4
35	2	1	3
36	1	1	2
37	3	1	4
38	1	1	2
39	1	1	2
Total	67	53	120

***Data are from Brasil et al. (*1*). APO, adverse pregnancy outcome.**

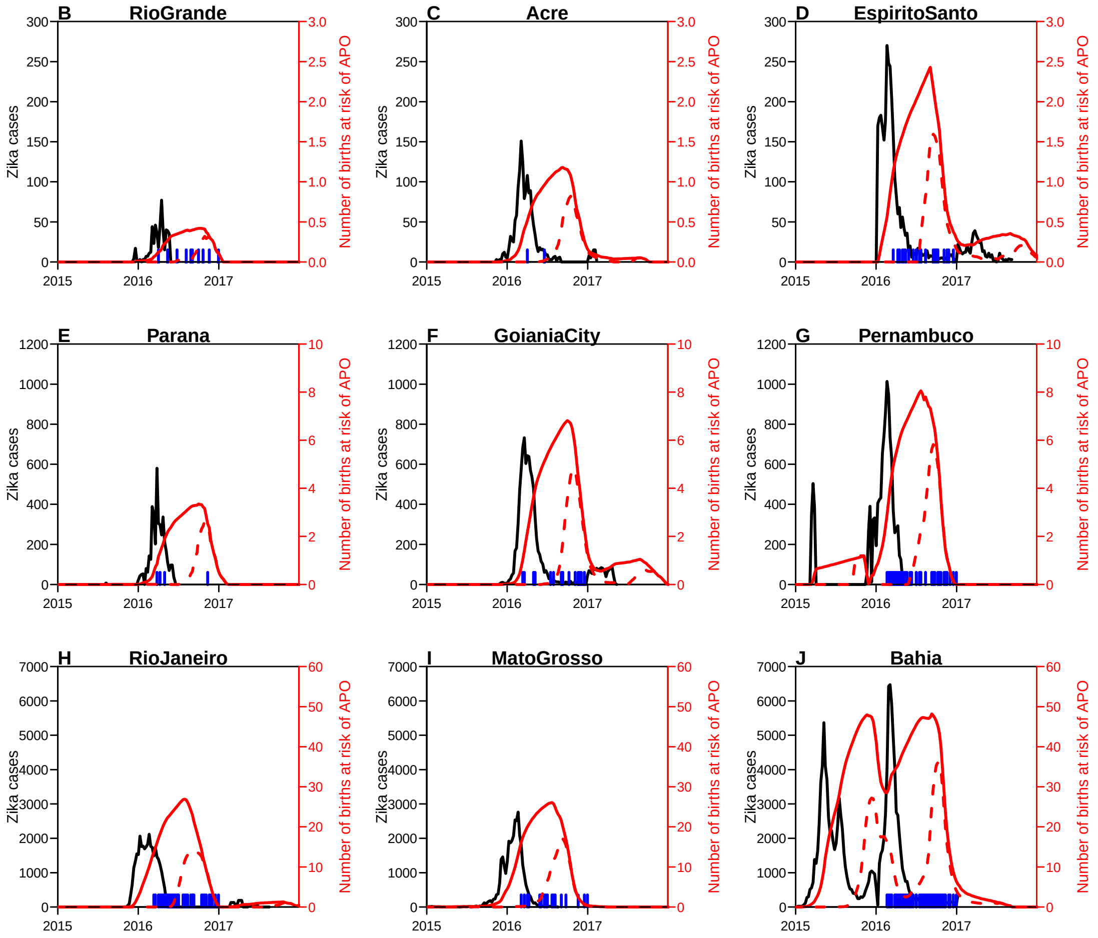

Technical Appendix Figure 1. Model-derived number and duration of adverse pregnancy outcomes (APOs) assuming 40% of Zika cases are reported, Brazil, April 2015–July 2017. Red line shows expected number of Zika-associated APO births from the fitted model (Figure 1, panel A main text), with 95% CI given by the shaded region for an assumption of 40% of Zika cases reported. Dashed line shows fixed risk in first trimester only. Black line shows suspected Zika cases in different regions. We assume 40% of Zika infections are reported in this sensitivity analysis. Blue ticks mark weeks in which microcephaly cases were reported in each region.

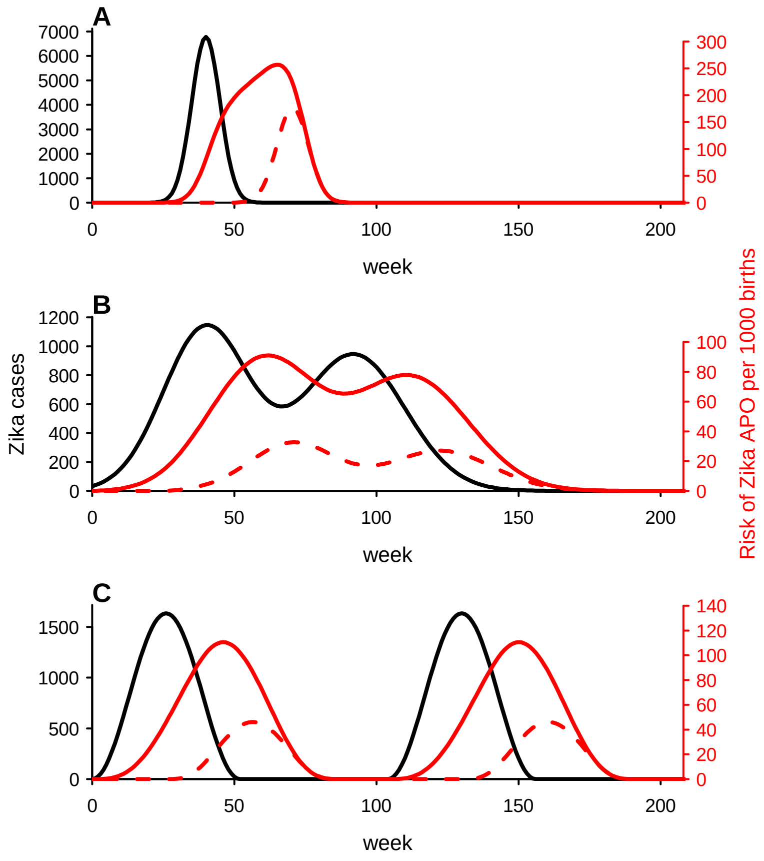

Technical Appendix Figure 2. Expected temporal distribution of adverse pregnancy outcomes (APOs) under different hypothetical outbreak scenarios, Brazil, April 2015–July 2017. Black line shows Zika cases (left axis); red shows expected proportion of births with Zika-associated APO in subsequent weeks based on the 2 risk distributions in A (right axis). A) Short, single-peaked outbreak. B) Double-peaked outbreak. C) Biennial epidemics (i.e., a seasonal endemic state). We assume a population size of 1 million, that 40% of Zika infections are reported, and a 50% attack rate during a 4-year period. APO, adverse pregnancy outcome.

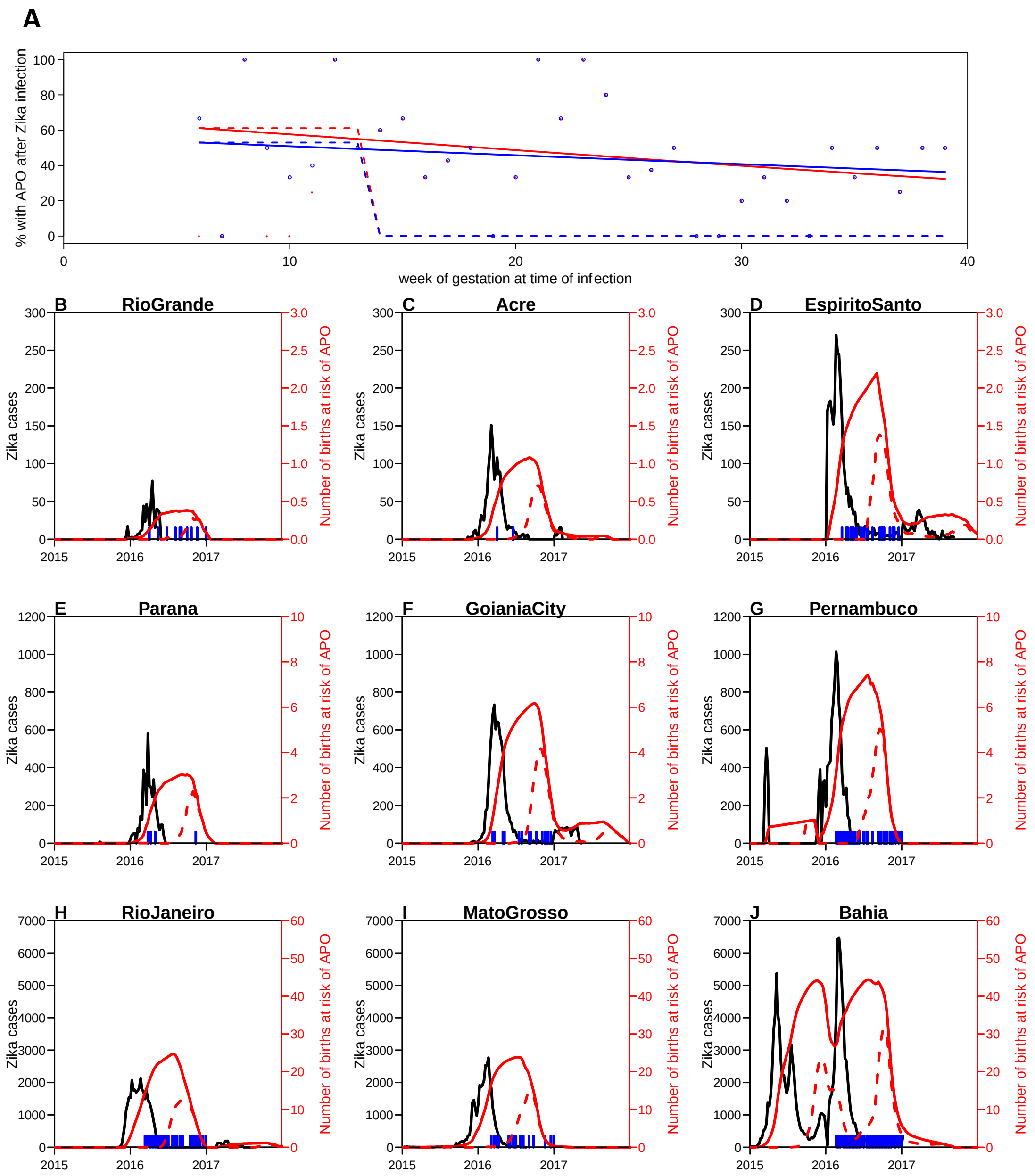

Technical Appendix Figure 3. A) Comparison of findings with and without miscarriage (fetal loss in first trimester) as an adverse pregnancy outcome (APO), Brazil, April 2015–July 2017. Red line shows fit to data including miscarriages and blue excludes those APOs, with 95% CI given by the shaded region. Dashed line shows fixed risk in first trimester only. B–J) Black line shows suspected Zika cases in different regions; red lines show expected number of births with Zika-associated APO in subsequent weeks based on the blue fitted risk distribution in Technical Appendix Figure 3, panel A. Blue ticks mark weeks in which microcephaly cases were reported in each region.
